# The immune checkpoint inhibitor avelumab increases aortic inflammation on [^18^F]FDG PET/CT: A retrospective cohort study

**DOI:** 10.1371/journal.pone.0339671

**Published:** 2025-12-29

**Authors:** Anniek Strijdhorst, Reindert F. Oostveen, Mark P.A. Schilder, Arthur J.A.T. Braat, Youssef Chahid, Damini Dey, Nordin M.J. Hanssen, Hanneke W.M. van Laarhoven, Anne W. van Schijndel, Tom T.P. Seijkens, Piotr J. Slomka, Erik S.G. Stroes, Margot Tesselaar, Hein J. Verberne, Nick van Es

**Affiliations:** 1 Amsterdam University Medical Center, location University of Amsterdam, Department of Vascular Medicine, Amsterdam, The Netherlands; 2 Cancer Center Amsterdam, Cancer Treatment and Quality of Life, Amsterdam, The Netherlands; 3 Amsterdam Cardiovascular Sciences, Atherosclerosis & Ischemic Syndromes, Amsterdam, The Netherlands; 4 Department of Nuclear Medicine, The Netherlands Cancer Institute—Antoni van Leeuwenhoek, Amsterdam, The Netherlands; 5 Department of Radiology and Nuclear Medicine, Amsterdam UMC, University of Amsterdam, Amsterdam, The Netherlands; 6 Department of Medicine (Division of Artificial Intelligence), Cedars-Sinai Medical Center, Los Angeles, California, United States of America; 7 Amsterdam University Medical Center, location University of Amsterdam, Department of Medical Oncology, Amsterdam, the Netherlands; 8 Department of Intensive Care, The Netherlands Cancer Institute—Antoni van Leeuwenhoek, Amsterdam, The Netherlands; 9 Department of Cardiology, The Netherlands Cancer Institute—Antoni van Leeuwenhoek, Amsterdam, the Netherlands; 10 Division of Medical Oncology, The Netherlands Cancer Institute—Antoni van Leeuwenhoek, Amsterdam, the Netherlands; Fondazione Policlinico Universitario Agostino Gemelli IRCCS, ITALY

## Abstract

**Background:**

Patients with cancer treated with immune checkpoint inhibitors (ICIs) are at increased risk of cardiovascular events. Preclinical studies suggest that this may result from inflammation-induced destabilization of atherosclerotic plaques.

**Objective:**

To evaluate changes in vessel wall inflammation assessed using [^18^F]FDG positron emission tomography/computed tomography (PET/CT) after ICI initiation.

**Methods:**

This was a single-center retrospective cohort study of patients with Merkel cell carcinoma who received at least one cycle of the programmed death ligand 1 (PD-L1) inhibitor avelumab and underwent [^18^F]FDG PET/CT before initiation of treatment and after 3 months. The primary outcome was the change in the target-to-background ratio (TBR_max_) in the descending aorta between baseline and first follow-up scan. Secondary outcomes included the change in TBR_max_ in the carotid arteries, spleen, and bone marrow, and incidence of major adverse cardiovascular events.

**Results:**

Fifty-three patients were included (66% male; median age 75 years). Most patients had established risk factors for cardiovascular disease (62%). The [^18^F]FDG TBR_max_ in the descending aorta increased from 1.52 (IQR, 1.39–1.70) at baseline to 1.64 (IQR, 1.41–1.97) after 3 months of treatment (change 7.8%. p = 0.022). No significant changes were observed in the carotid arteries, bone marrow, and spleen. Statin use was not associated with an attenuated change in TBR_max._ During a median follow-up of 2.3 (IQR, 1.5–4.2) years, one nonfatal ischemic stroke occurred.

**Conclusion:**

Avelumab treatment was associated with an increase in [^18^F]FDG uptake in the descending aorta after 3 months of treatment, which may be a potential marker of inflammation-driven accelerated atherosclerosis in patients receiving ICIs.

## Introduction

Immune checkpoint inhibitors (ICI) have revolutionized cancer treatment by significantly increasing overall survival in many types of cancer [1]. Monoclonal antibody-mediated inhibition of co-inhibitory proteins located on T cells elicits T cell-mediated anti-tumor immune responses. While ICI enhances the cellular immune response against tumors, it has also been shown to potentially induce T cell mediated adverse effects such as thyroiditis, hepatitis, and colitis [2].

Besides these well-characterized acute side effects, emerging data demonstrate that patients receiving ICI are hallmarked by a 2- to 3-fold increased risk of cardiovascular events, predominantly myocardial infarction and ischemic stroke [3,4]. The underlying pathophysiology has been suggested to relate to an adverse impact of the systemic pro-inflammatory effect of ICI on pre-existing atherosclerotic plaques leading to destabilization and an increased rupture risk [5]. Studies in mice have demonstrated that inhibition of the immune checkpoint proteins cytotoxic T-lymphocyte-associated protein 4 (CTLA-4) and programmed death-1 (PD-1) increases atherosclerotic lesion size, accompanied by an inflammatory plaque phenotype with enhanced infiltration of T cells and monocytes into the subendothelial space, leading to a pro-inflammatory milieu in atherosclerotic plaques [6–8].

Imaging has been used to non-invasively assess changes in atherosclerotic plaques in patients receiving ICI. Small studies using computed tomography (CT) have shown a 3-fold increase in atherosclerotic aortic plaque volume one year after the start of ICI [3,9]. However, changes in plaque volume do not necessarily reflect changes in vascular inflammation. Functional imaging with positron emission tomography CT (PET/CT) could provide valuable insights into whether immune checkpoint inhibitors elicit a pro-inflammatory state in atherosclerotic plaques in patients. The glucose analogue 2-deoxy-2[^18^F]fluoro-D-glucose ([^18^F]FDG) is a commonly used tracer that detects an increased metabolic activity, which in the case of the arterial wall has been attributed largely to increased metabolic activity of inflammatory cells, predominantly macrophages [10]. Several large retrospective studies have demonstrated that vascular [^18^F]FDG uptake can help identify individuals at high risk of cardiovascular events, while it has also been established as a surrogate marker for residual inflammatory risk in intervention studies [11,12].

Therefore, the objective of this study was to evaluate changes in arterial inflammation after initiation of ICI using [^18^F]FDG PET/CT imaging. We used data from a retrospective cohort of patients with metastatic or irresectable Merkel cell carcinoma treated with the programmed death-ligand 1 (PD-L1) avelumab [13].

## Methods

### Study design and study group

This was a single-center retrospective cohort study conducted in the Antoni van Leeuwenhoek hospital, a referral center for the treatment of Merkel cell carcinoma, a rare cancer of the skin. Patients were eligible for inclusion if they had a confirmed diagnosis of metastatic or irresectable Merkel cell carcinoma, received at least one cycle of the PD-L1 inhibitor avelumab, and underwent a ^18^F-FDG PET/CT before the initiation of avelumab and at least one scan after initiation, between July 1, 2017, and November 30, 2023. Patients were identified using the hospital’s electronic health records. Avelumab 600 mg was given every 2 weeks until disease progression, intolerable side effects, or death. The most recent PET/CT performed prior to the start of avelumab treatment was designated the baseline scan (T0). As per local protocol, the first evaluation scan was performed approximately 3 months after treatment initiation (T1). The following data were collected from the electronic health records: age, sex, risk factors for cardiovascular disease (i.e., obesity, hypertension, dyslipidemia, diabetes mellitus, and smoking), medication (i.e., prior chemotherapy, lipid lowering therapy, glucose lowering agents, antihypertensive drugs, anticoagulation, antiplatelet therapy, and immunosuppressive agents), previous cardiovascular events, immune-related adverse events, major cardiovascular events, and all-cause death. Patients were followed until death, loss to follow-up, or last date of data collection (January 3, 2025). Hypertension was defined as the use of blood pressure lowering medication. Dyslipidemia was defined as the use of lipid lowering therapy. The study protocol was approved by the Investigational Review Board of the Netherlands Cancer Institute (NKI) (Approval number IRB23–288) and all patients provided informed consent. This report adheres to the Strengthening of Reporting of Observational Studies (STROBE) statement (see checklist in S1 File).

### Image acquisition and reconstruction

PET/CT scans were performed on a Philips Gemini TF 16 or the Philips Gemini TF big-bore PET/CT scanner (Philips, Cleveland, USA) using 3.5 MBq/kg [^18^F]FDG activity. Patients were required to fast for a minimum of 4 hours prior to imaging. In addition, patients were instructed to drink at least 500 mL of fluids prior to the scan. Imaging was performed approximately 60 minutes after tracer administration. The total scan time varied depending on the number of bed positions, with each bed position being imaged for 1–3 minutes. The scan coverage was tailored based on the primary tumor localization and was either from skull base to the inguinal region, or total body. A low-dose, non-contrast CT scan was performed with the following parameters: 40 mAs, 140 kV, and slice thickness ranging from 2 to 5 mm. Routine clinical PET image reconstruction was used for standardized uptake value (SUV) measurements.

### Image analysis

The analysis of [^18^F]FDG PET/CT scans was conducted using specialized research software (FusionQuant, Cedars-Sinai Medical Center, CA, USA) by MS and RFO. The assessors were blinded to patient information, as all scans were anonymized and assigned a random study ID. To evaluate the uptake of [^18^F]FDG in the aorta and carotid artery, we employed the maximum target-to-background ratio (TBR_max_), which is calculated by dividing the maximum standardized uptake value (SUV_max_) of the target tissue by the SUV_mean_ of the background blood in the superior vena cava (SVC) [13,25]. For measuring tracer uptake in the carotid artery, a region of interest (ROI) with a diameter of 4 mm was positioned around the right common carotid artery, extending from the division of the brachiocephalic artery to the bifurcation. For the analysis of the descending aorta, the ROI diameter was set to the aortic wall diameter plus an additional 4 mm to accommodate spatial resolution. The descending aorta was assessed from the apex of the descending segment until the spleen appeared in the transverse plane of the CT scan.

For assessing bone marrow and splenic uptake we adopted methods as published previously [14]. In short, the SUV_max_ in the spleen was evaluated by outlining a cylindrical volume of interest (VOI) with a 5 mm diameter around the region of highest uptake. The bone marrow SUV_max_ was calculated by placing a VOI across six thoracic vertebrae, with the reported value representing the average SUV_max_ of these vertebrae. All SUV calculations were according to a body weight corrected formula.

### Study outcomes

The primary outcome was the change in [^18^F]FDG (expressed as TBR_max_) uptake in the descending aorta between the baseline (T0) and first follow-up scan (T1). Secondary outcomes included the change in [^18^F]FDG uptake in the carotid arteries, spleen, and bone marrow. Uptake in spleen and bone marrow indicates hematopoietic activity. We also assessed the incidence of major cardiovascular events, defined as the composite of nonfatal ischemic stroke, nonfatal myocardial infarction, and cardiovascular death, and immune related adverse events (irAE) graded according to Common Terminology Criteria for Adverse Events, version 5.0 [13].

### Statistical analysis

Standard descriptive statistics were used. Change in TBR_max_ between T0 and T1 was assessed by the Wilcoxon signed-rank test. Factors associated with change in TBR_max_ in the descending aorta were evaluated using linear regression models. All baseline variables were first evaluated for association with TBR_max_ in an univariable regression analysis, and only variables associated with TBRmax at P ≤ 0.20 were subsequently included in the exploratory multivariable model. We also assessed the potential effect of statins on arterial inflammation, as previous studies suggested a protective role of these drugs [15–17]. Long-term changes in [^18^F]FDG uptake in the aorta were explored in patients with at least four PET/CT scans by performing a trend analysis. Two-sided P-values below 0.05 were considered to indicate statistical significance. No sample size calculation was performed; all eligible patients between July 2017 and November 2023 were included. All statistical analyses were performed in R, version 4.3.2 (www.R-project.org), and Figures were created with GraphPad Prism version 10.0.0 for Windows (GraphPad Software, Boston, Massachusetts USA, www.graphpad.com).

## Results

Between July 2017 and November 2023, 62 patients with metastatic Merkel cell carcinoma (MCC) and 7 with locally advanced irresectable MCC received at least one cycle of first-line treatment with avelumab of whom 53 (77%) had both baseline and follow-up scans. The median age was 75 years (interquartile range [IQR], 70–81) and 66% of patients were male (see **Table 1** for baseline characteristics). The majority of patients had one or more established risk factors for cardiovascular disease (62%). The median number of completed cycles at three months was 7. There was no difference in median plasma glucose between baseline (5.8 mmol/L) and 3-month follow-up (5.9 mmol/L). During the median follow-up period of 2.3 (IQR, 1.5–4.2) years, eighteen patients (34%) died and seven (13%) were lost to follow-up after the second PET/CT scan was performed due to treatment in other hospitals or best supportive care after progressive disease.

**Table 1:** Baseline characteristics.

	Cohort (n = 53)
Median age (range) – years	75 (70, 81)
Male sex – no. (%)	35 (66%)
History of cardiovascular disease – no. (%)	
Myocardial infarction	7 (13%)
Coronary revascularization	8 (15%)
Ischaemic stroke	1 (2%)
Transient ischaemic attack	1 (2%)
Peripheral artery disease	1 (2%)
Cardiovascular risk factors – no. (%)	
Hypertension^*^	24 (45%)
Hypercholesterolemia^#^	23 (43%)
Diabetes mellitus	12 (36%)
BMI ≥ 30 kg/m2	14 (26%)
Smoking^$^	23 (43%)
Auto-immune disease	6 (11%)
Medication use	
Anticoagulants	4 (8%)
Platelet aggregation inhibitors	12 (23%)
Immunosuppressive drugs	
Prednisone	5 (9%)
Colchicine	3 (6%)

Percentages may not total 100 because of rounding. Abbreviations: no., number; BMI, body mass index.

* Hypertension was defined as the use of blood pressure lowering therapy.

# Hypercholesterolemia was defined as the use of lipid lowering therapy.

$ Smoking was defined as prior or active smoking.

### Imaging results

**Fig 1:**
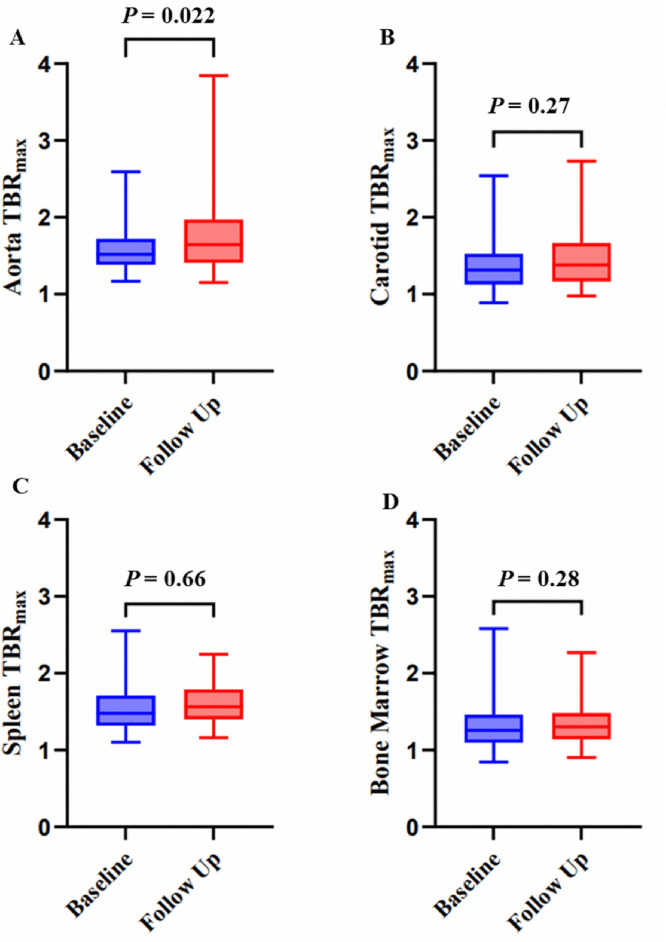
Change in [^18^F]FDG uptake between baseline and follow-up PET-CT. (A) Descending aorta. (B) Carotid arteries. (C) Spleen. (D) Bone marrow.

The median time between the baseline (T0) and first follow-up scan (T1) was 120 days (IQR, 101−132), and the median time between the start of avelumab and T1 was 90 days (IQR, 78−107). There was a significant increase in TBR_max_ in the descending aorta from 1.52 (IQR, 1.39–1.70) at T0 to 1.64 (IQR, 1.41–1.97) at T1 (p = 0.022) (Fig 1). No difference was observed in the TBR_max_ in the carotid arteries between T0 and T1 (1.31 vs 1.37; p = 0.27). Also, no change in splenic TBR_max_ (1.48 vs 1.57; p = 0.66) and bone marrow TBR_max_ was observed (1.26 vs 1.31; p = 0.28). Results were similar when patients who were treated with glucocorticoid between T0 and T1 because ofirAEs were excluded. In a subgroup analysis of patients who completed all 7 cycles between baseline and the follow-up PET/CT at 3 months, similar results were found for the increase in aortic TBR_max_ (p = 0.01, S1 Table)

### *Factors associated with changes in TBR*_*max*_

Between T0 and T1, six patients (11%) developed grade ≥3 irAE for which they received immunosuppressive agents. Development of irAE was not associated with a change in aortic TBR_max_ (p = 0.72). In univariable linear regression analysis, age was significantly associated with a reduction in TBR_max_ between T0 and T1 at P < 0.05, while body mass index was associated with a reduction in TBR_max_ at P < 0.20 (**Table 2**). These variables were then included in a multivariable linear regression in which age remained negatively associated with change in TBR_max_ at P < 0.05. Pre-existing statin use was not associated with a change in TBR_max_ following avelumab initiation after adjusting for age, sex, and baseline TBR_max_ (P = 0.58). Subgroup analysis did not reveal a significant difference in the change in TBR_max_ uptake between smokers and non-smokers (p = 0.81) nor between patients with and without type 2 diabetes (p = 0.88) (S1 Table).

**Table 2:** Univariable and multivariable regression model of baseline predictors on ΔTBR_max_ in the descending aorta.

Variable	Univariable analysis		Multivariable analysis	
	β (95% CI)	p-value	β (95% CI)	p-value
Age	−0.023 (−0.037, −0.001)	0.002	−0.023 (−0.036, −0.011)	<0.001
Sex (female)	−0.934 (−0.393, 0.207)	0.545		
Use of blood pressure medication	−0.023 (−0.401, 0356)	0.907		
Use of lipid lowering therapy	−0.176 (−0.459, 0.108)	0.230		
Diabetes mellitus type 2	0.062 (−0.287, 0.412)	0.728		
Body mass index	−0.020 (−0.047, 0.008)	0.170		
Smoking^$^	0.249 (−0.328, 0.825)	0.403	−0.021 (−0.04, −0.002)	0.079
Immune suppressive use at baseline	−0.073 (−0.522, 0.377)	0.753		

$ Smoking was defined as prior or active smoking.

* Immune related adverse events grade ≥3

Abbreviations: TBR_max_, maximum target-to-background ratio

### *Long term changes in TBR*_*max*_

Eight patients underwent four or more PET/CT scans for disease monitoring during follow-up. The median intervals between baseline (T0) and the third (T2) and fourth follow-up scans (T3) were 6 and 9 months, respectively. TBR_max_ at 9 months appeared to be higher than at baseline, but no further increase was observed thereafter (P_trend_ = 0.27; S1 Fig).

### Cardiovascular events

During the median follow-up period of 2.3 (IQR, 1.5–4.2) years, one (2%) patient developed a nonfatal ischemic stroke. Four patients developed non-major cardiovascular events including a transient ischemic attack (n = 2; 4%), below-knee amputation due to ischemic peripheral artery disease (n = 1; 2%), and percutaneous transluminal angioplasty and stent placement for a stenosis in an aorto-bifurcated prosthesis (n = 1; 2%). Change in TBR_max_ between T0 and T1 was not associated with major or non-major cardiovascular events (p= 0.85).

## Discussion

Using [^18^F]FDG PET/CT, the PD-L1 immune checkpoint inhibitor avelumab was associated with an increase in aortic TBR_max_ within 3 months after treatment initiation in patients with metastatic or irresectable Merkel cell carcinoma. These findings support the concept that systemic immune checkpoint inhibition results in vascular inflammation, which may contribute to the increased risk of cardiovascular events observed in patients receiving immune checkpoint inhibitors.

Atherosclerosis is characterized by chronic low-grade inflammation of the arterial wall, in which T cells are the most abundant immune cells present in the atherosclerotic plaques [18,19]. Murine studies have demonstrated that ICI leads to pro-inflammatory changes in the plaques, characterized by an increase in CD4^+^ and CD8^+^ T cells, macrophage content, and a larger necrotic core [6,7,20]. Upregulation of the adhesion molecules ICAM-1 and VCAM-1 on the endothelial lining was also observed, confirming activation of the endothelium with enhanced recruitment of immune cells to the atherosclerotic plaques [7,20,21]. An adverse effect of inhibition of immune checkpoint proteins on atherogenesis is further supported by a decrease in plaque size and more stable phenotype when, conversely, CTLA-4 and PD-1 are stimulated [22–24]. To our knowledge, no other studies to date have evaluated the effects of a PD-L1 inhibitor, specifically avelumab, on arterial inflammation.

The significant increase in aortic TBR_max_ after 3 months of treatment with avelumab in patients with metastatic or irresectable Merkel cell carcinoma aligns with previous studies. In a retrospective cohort study of 96 melanoma patients, Polomski and colleagues found a significant increase in [^18^F]FDG uptake in large arteries was observed within the first 6 months of ICI treatment, while no further increase was observed beyond 6 months [15]. Three other small retrospective cohort studies also observed increased [^18^F]FDG uptake in large arteries within 6–9 months of treatment with PD-1 and/or CTLA-4 inhibitors [14,23,24]. In contrast, a small PET-imaging pilot study [7] including 10 relatively young melanoma patients without a history of cardiovascular disease did not find a difference in FDG uptake after 6 weeks of PD-1 and/or CTLA-4 treatment. This population may not have been at high risk for accelerated atherosclerosis, and the short interval between scans may have limited the ability to detect changes in vascular inflammation. We did not observe changes in TBR_max_ beyond 3 months in eight patients with multiple PET/CT scans during follow-up, suggesting that the pro-inflammatory effects of ICIs are most pronounced in the first months after initiation of treatment [17]. However, a recent study by Bacmeister and colleagues found an significant increase in FDG uptake of 2.5% annually up to 2 years after initiation of ICI [15]. This discrepancy underscores the need for longitudinal studies to determine the long-term vascular effects of ICI.

We focused on an elderly population with metastatic or irresectable Merkel cell carcinoma. These patients may have different characteristics than younger cancer patients, and a higher proportion may have pre-existent atherosclerotic plaques with a different phenotype. In the risk factor analysis, we found that age was negatively correlated with change in TBR_max_, also when adjusted for baseline TBR_max_. Whether this inverse association is causal or due to residual confounding is unclear. A potential explanation may be that elderly patients with extensive vascular calcifications exhibit a smaller [^18^F]FDG increase due to reduced baseline inflammatory activity of plaques, as shown in previous studies in patients who received ICI [17,25]. This is in line with data demonstrating that calcified plaques have less [^18^F]FDG uptake than metabolically active lesions [26]. Further studies are needed to assess the association of increased arterial [^18^F]FDG uptake and the incidence of cardiovascular events in patients receiving ICI.

In contrast to the increase in TBR_max_ in the descending aorta, we found no changes in carotid TBR_max_ after the initiation of avelumab. The images were obtained from scanners with limited spatial resolution, which hampered accurate carotid measurements on non-contrast enhanced scans. However, this finding may also reflect low baseline carotid plaque inflammation in this cohort. Notably, we also observed no significant changes in [^18^F]FDG uptake in the spleen or bone marrow, consistent with findings from Calabretta et al., suggesting that avelumab-induced vascular inflammation is not merely the result of increased hematopoietic activity [16].

Several limitations of this study should be acknowledged. First, the sample size was small because Merkel cell carcinoma is a rare tumor type, which limited statistical power. Second, coronary arterial inflammation could not be assessed since the [^18^F]FDG PET/CT scans were not cardiac-gated. Third, only eight patients had multiple follow-up scans available hampering the analysis of long-term changes. Fourth, the European Association of Nuclear Medicine (EANM) recommends acquiring PET images 2 hours after tracer injection to ensure reliable quantification of arterial wall FDG uptake, and prescan blood glucose levels below 130 mg/dL [27]. In our retrospective study, PET/CT scans were acquired for oncological purposes, with image acquisition performed between 52 and 74 minutes after tracer injection, which may not have been optimized for assessing tracer uptake in the arterial wall. Additionally, 80% of patients at baseline and 87% at 3 months had prescan glucose levels below 130 mg/dL, and there was no significant difference in glucose levels between scans (p = 0.71). While these factors may have introduced some variability, the majority of scans met the recommended glucose threshold, and the timing reflects standard clinical practice for oncology patients. Fifth, as this was a retrospective study with considerable loss to follow-up, outcome events may have been missed.

The observed increase in ^18^FDG uptake in large arteries in elderly patients with metastatic or irresectable Merkel cell carcinoma receiving avelumab supports the concept of ICI-induced arterial wall inflammation, which has been shown to lead to plaque destabilization in murine studies. Although our sample size was too small to evaluate the association between changes in arterial wall inflammation and cardiovascular events, previous studies have demonstrated that arterial wall inflammation predicts major cardiovascular events [10]. Therefore, with the expanding use of ICIs, also in the neoadjuvant or adjuvant setting, identifying patients at high risk for cardiovascular events is becoming increasingly relevant. The European Society of Cardiology (ESC) Cardio-Oncology guidelines advocate routine cardiovascular assessment every 6–12 months in patients requiring ICI for more than one year, with more intensive surveillance recommended for those classified as being at high risk of CVD [28]. However, this recommendation ignores the increased risk of major cardiovascular events observed shortly after starting ICI in other studies [3,4], and it does not provide guidance on the optimal risk stratification nor risk mitigation strategies.

Whether arterial wall inflammation detected by PET/CT-scans could serve as a biomarker for cardiovascular risk in this setting remains an open question, requiring future larger studies. The observed changes in FDG uptake after ICI initiation suggest that this imaging marker, reflecting macrophage activity, could be valuable in future studies investigating risk mitigation strategies. Previous studies have shown that increased vascular FDG uptake is associated with a increased risk for ASCVD [29]. Therefore, future studies should evaluate whether and how these routinely available PET/CT data can be used to better identify patients at high risk of ASCVD. In addition, FDG PET or newer tracers such as DOTATATE [30] may help to evaluate the effect of interventions aimed at reducing vascular inflammation in this population.

Exploring alternative imaging modalities, such as coronary CT angiography, may offer a more accessible approach. Surrogate markers of arterial inflammation, like the fat attenuation index, strongly correlate with future cardiovascular events [31]. However, its integration into routine clinical practice remains challenging due to the need for dedicated ECG-gated scans. Furthermore, whether patients with ICI-induced arterial inflammation could benefit from prophylactic treatment with statins or anti-inflammatory therapies has not yet been studied.

Overall, our findings support previous concerns regarding the potential adverse cardiovascular effects of ICI. As ICI use expands, future research should determine whether PET/CT-detected arterial inflammation can predict cardiovascular events and whether alternative imaging techniques could provide a more practical risk assessment tool. Investigating targeted interventions to mitigate ICI-induced cardiovascular disease will be crucial to optimizing cardiovascular outcomes in this patient population.

## Supporting information

S1 Table Subgroup analyses of the change in TBRmax in the descending aorta between baseline and 3 months, stratified by cardiovascular risk factors and treatment characteristics. (DOCX)

S1 Fig Change in TBR_max_ in the descending aorta over time in eight patients with multiple follow-up scans. Timepoints: T0, baseline; T1, + /- 3 months; T2, + /- 6 months; T3, + /- 9 months. Abbreviations: TBR_max_, target-to-background ratio.
(TIF)

S1 File STROBE checklist. (PDF)
