# Supplementary material for: The immune checkpoint inhibitor avelumab increases aortic inflammation on [18F]FDG PET/CT: A retrospective cohort study
Source: PLoS One. 2025 Dec 29;20(12):e0339671. doi: 10.1371/journal.pone.0339671 (PMC12747342; doi:10.1371/journal.pone.0339671)
Supplement: S1 Table — (DOCX) [file pone.0339671.s001.docx]

**Supplemental Table 1.** Subgroup analyses of the change in TBR_max_ in the descending aorta between baseline and 3 months, stratified by cardiovascular risk factors and treatment characteristics.

| Variable | Group | n | ΔTBR_max,_ median (IQR) | *P* value |
| --- | --- | --- | --- | --- |
| Smoking history | Yes | 23 | 0.21 (-0.12-0.42) |  |
|  | No | 30 | 0.04 (-0.12-0.42) | 0.81 |
| Diabetes mellitus type 2 | Yes | 12 | 0.16 (-0.09-0.43) |  |
|  | No | 41 | 0.08 (-0.12-0.39) | 0.88 |
| Completed avelumab cycles | 7 | 48 | 0.12 (-0.09-0.45) |  |
|  | <7 | 5 | -0.27 (-0.42-0.19) | 0.15 |

ΔTBRmax is calculated as the change in TBR_max_ in the descending aorta between baseline and 3 months. Abbreviations: n, number.
