# Supplementary figures and images for: The immune checkpoint inhibitor avelumab increases aortic inflammation on [18F]FDG PET/CT: A retrospective cohort study
Source: PLoS One. 2025 Dec 29;20(12):e0339671. doi: 10.1371/journal.pone.0339671 (PMC12747342; doi:10.1371/journal.pone.0339671)

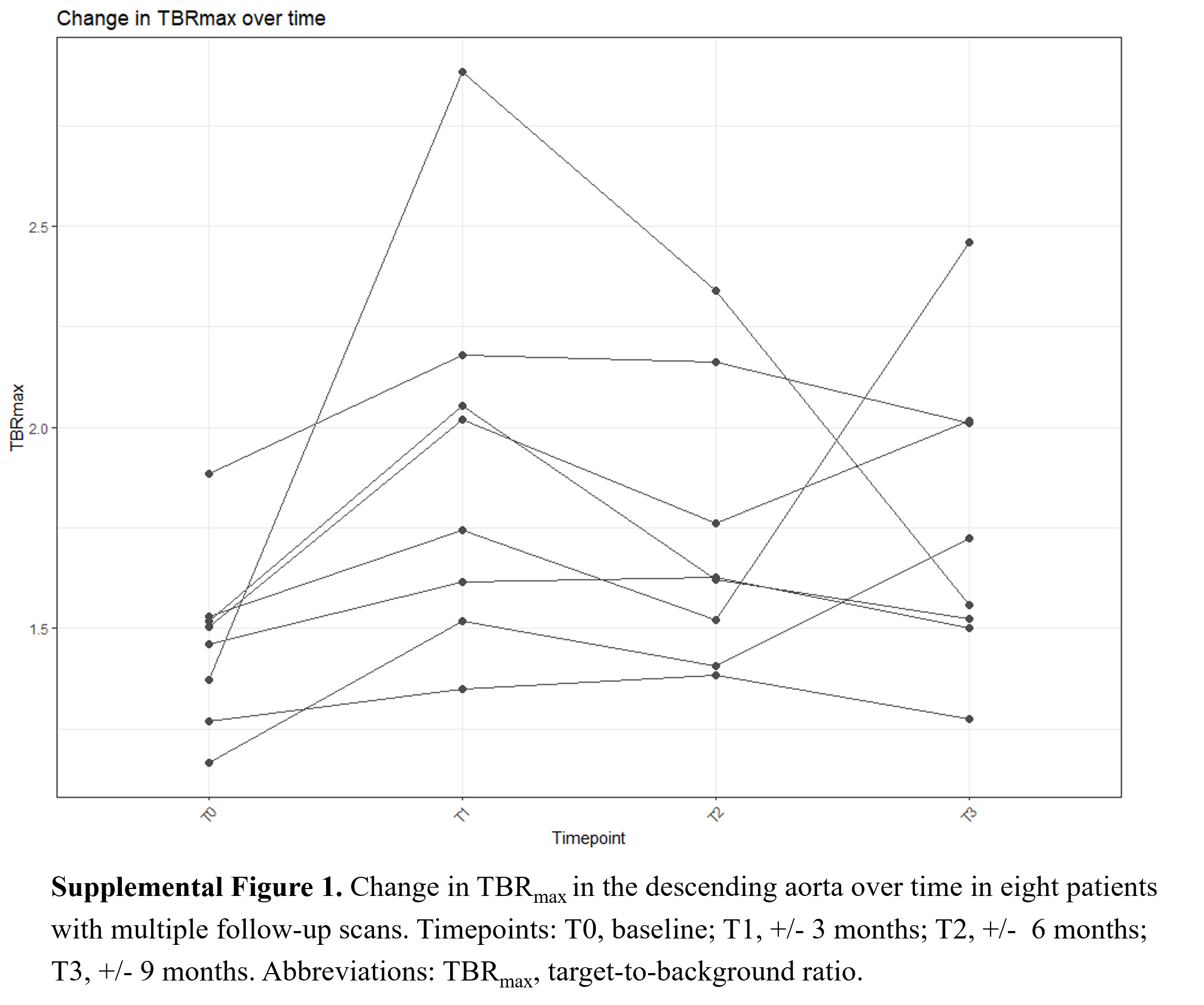

Supplement: S1 Fig — Timepoints: T0, baseline; T1, + /- 3 months; T2, + /- 6 months; T3, + /- 9 months. Abbreviations: TBRmax, target-to-background ratio. (TIF) [file pone.0339671.s002.tif]
